# Toxicological Effects of Microplastics and Sulfadiazine on the Microalgae *Chlamydomonas reinhardtii*

**DOI:** 10.3389/fmicb.2022.865768

**Published:** 2022-04-28

**Authors:** Ze Li, Sheng Dong, Fei Huang, Langli Lin, Zhangli Hu, Yihong Zheng

**Affiliations:** ^1^Guangdong Provincial Key Laboratory for Plant Epigenetics, Guangdong Engineering Research Center for Marine Algal Biotechnology, College of Life Sciences and Oceanography, Shenzhen University, Shenzhen, China; ^2^Shenzhen Engineering Laboratory of Microalgal Bioenergy, Harbin Institute of Technology Shenzhen, Shenzhen, China

**Keywords:** microplastics size, antibiotics, algae, combined toxicity, oxidative stress

## Abstract

Despite the fact that microplastics (MPs) facilitate the adsorption of environmental organic pollutants and influence their toxicity for organisms, more study is needed on the combination of MPs and antibiotics pollutant effects. In this study, polystyrene MPs (1 and 5 μm) and sulfadiazine (SDZ) were examined separately and in combination on freshwater microalga, *Chlamydomonas reinhardtii*. The results suggest that both the MPs and SDZ alone and in combination inhibited the growth of microalgae with an increasing concentration of MPs and SDZ (5–200 mg l^–1^); however, the inhibition rate was reduced by combination. Upon exposure for 7 days, both the MPs and SDZ inhibited algal growth, reduced chlorophyll content, and enhanced superoxide dismutase (SOD) activities, whereas glutathione peroxidase (GSH-Px) activity was elevated only with the exposure of 1 μm MPs. Fluorescence microscopy and scanning electron microscopy also indicated that particle size contributed to the combined toxicity by aggregating MPs with periphery pollutants. Further, the amount of extracellular secretory protein increased in the presence of MPs and SDZ removal ratio decreased when MPs and SDZ coexisted, suggesting that MPs affected SDZ metabolism by microalgae. The particle size of microplastics affected the toxicity of MPs on microalgae and the combined effect of MPs and SDZ could be mitigated by MPs adsorption. These findings provide insight into microalgae responses to the combination of MPs and antibiotics in water ecosystems.

## Introduction

The invention and use of plastic products have made human life more convenient; in spite of all these good feathers, plastic pollution has become a serious major global environmental threat because of its physicochemical stability and difficulty in degrading naturally in the environment, especially in the aquatic ecosystems (Barboza et al., 2018; Zhu et al., 2019). The effects of plastic pollution in the aquatic environment are being continually discovered, with the presence of plastic components has been detected in the ocean even at depths of 7,000–11,000 m and the discharge of plastic waste is increasing every year (Zhu et al., 2019). The toxic chemical additives are leaching in aqueous environment due to the plastic contaminants; furthermore, they also provide adsorption capacity for the enrichment of antibiotic-resistant bacteria and pesticide residues, which resulting in severe effects on marine ecosystems (Chae et al., 2019; Zhang et al., 2020), riverine ecosystems (Zhang et al., 2018), and lake ecosystems (Beiras et al., 2021). Known as microplastics (MPs), these plastic pellets with a diameter ranging from 0.1 and 5 mm are long-term environmental contaminants (Sun et al., 2020). These MPs accumulate in aquatic animal through the food chain lead to affect their growth and development, reducing their nutritional status and harmful to the ecosystems and posing a health threat to humans (Coyle et al., 2020; Elizalde-Velázquez and Gómez-Oliván, 2021). In addition, the mixing of multiple pollutants further exacerbates the contamination of MPs due to its roughness, porosity, polarity, and hydrophobicity (Brandon et al., 2016; Nava and Leoni, 2021); it enables MPs to adsorb more contaminants in the environment—heavy metals, antibiotics, persistent organic pollutants, and other contaminants (Holmes et al., 2012; Wright et al., 2013; Coyle et al., 2020). As primary producers of aquatic ecosystems, microalgae may be affected by the toxicity of MPs pollution. In addition to the effects on microalgal growth, studies show that MPs affect algal photosynthesis, as chlorophyll content and photosynthesis efficiency decrease with exposure to MPs and that smaller sizes are considered more toxic. However, study into the effects of mixing microplastics with different pollutants is scarce (Nava and Leoni, 2021). A joint toxicity study of 0.3 mg l^–1^ triclosan and four kinds of 50 mg l^–1^ MPs was conducted with the microalgae *Skeletonema costatum* and it suggests that aggregation affected their combined effect by reducing the total superoxide dismutase (SOD) enzyme activity (Zhu et al., 2019). In another study, combined effects of MPs and mercury (ppb scale) caused neurotoxicity and lipid oxidative damage of *Dicentrarchus labrax* (Barboza et al., 2018). Hence, it is essential to study the combined toxicity of MPs and other pollutants.

Antibiotics are important pharmaceutical and personal care products (PPCPs) and some of them are relatively stable and can persist in surface water and even drinking water, raising concerns about their potential dangers (Chaturvedi et al., 2021). Among them, sulfonamides are the earliest category of synthetic drugs with a broad antibacterial spectrum, definite efficacy, convenience, and safety and are widely used in aquaculture; however, the removal rate of sulfonamides is low in the conventional wastewater treatment process (Zhang et al., 2019). Moreover, as a heavily used group of veterinary antibiotics, sulfonamides have high mobility and low sorption affinity in soil, making them more likely to leach into groundwater (Rath et al., 2019). This has led to a rise in the level of this contaminant in the water. A recent study shown that the growth of *Chlorella vulgaris* (*C. vulgaris*) was inhibited with an increasing SDZ concentrations (10–270 mg l^–1^), which may be related to reactive oxygen species (ROS) damage to the algal photosynthetic system and chlorophyll biosynthesis. Furthermore, oxidative stress increases the activity of SOD and glutathione reductase, while decreases the activity of catalase. This made the antioxidant response inadequate to cope with the rising ROS and prevent oxidative damage (Chen et al., 2020a). Studies have shown that the co-occurrence of MPs and antibiotics decreases microbial activity and diversity in natural environments such as soil and nitrifying sludge, resulting in combined pollution (Wang et al., 2020a,b).

As a primary producer, microalgae affect the structure and function of an aquatic ecosystem. Microalgae are sensitive to toxic substances, so they are a promising indicator of microplastic pollution threats to freshwater ecosystems (Zhang et al., 2017). Microalgae have been considered to be sensitive to the ubiquitous MPs and antibiotics and studies have been focused on the effects of single pollutants (Prata et al., 2018; Machado and Soares, 2019). The studies on the combined toxicity of these two types of pollutants are still limited. For instance, polystyrene (PS)-MPs affected the removal of levofloxacin by altering the adsorption, enrichment, and enzymatic degradation of antibiotics by *Chlorella vulgaris*; the levofloxacin (initial concentration of 93.8 μg l^–1^) removal rates for the microplastics group (35 items⋅L^–1^) and the control group were 23.34 and 46.71%, respectively, on the third day, but the combined toxicity on microalgae was not extensively studied (Wu et al., 2022).

As a typical phytoplankton, *Chlamydomonas reinhardtii* (*C. reinhardtii*) has a great prospective to easy cultivation, considered as highly susceptible to environmental pollution, used as potential candidate for aquatic contamination assessments, and demonstrated high biosorption and removal efficiency of PPCPs (Xie et al., 2020). In this study, we evaluated the potential toxicity of MPs and sulfonamide antibiotics to *C. reinhardtii*, according to the effects on cell growth of the microalgae and the physiological and biochemical responses, as well as investigating whether microalgae secrete extracellular substances that defend against MPs and antibiotics by adhesion.

## Materials and Methods

### Materials

Virgin PS-MPs microspheres (1 and 5-μm diameter, Cat. No. 7-3-0100 and 7-3-0500, respectively), labeled with green fluorescence (excitation wavelength: 470 nm and emission wavelength: 526 nm), were purchased from BaseLine ChromTech Research Centre (Tianjin, China) and IR absorption spectra confirmed the chemical composition of the microspheres. The diameters of PS-MPs particle were detected using the scanning electron microscope (SCM). Sulfadiazine (SDZ) sodium salt was purchased from Sigma-Aldrich (Cat. No. S6387-25G and purity 99.9%).

### Algal Culture

*Chlamydomonas reinhardtii* CC124 strain was obtained from the *Chlamydomonas* Genetic Center of Duke University (Durham, North Carolina, United States) and cultured in a tris-acetate-phosphate (TAP) medium. Algal cells were cultivated in a constant temperature light incubator at 22 ± 2°C and 20 μmol photon m^–2^s^–1^ illumination. Algae were grown in 250 ml Erlenmeyer flasks and were shaken daily and randomly arranged to reduce any minor differences in photon irradiance (Huang et al., 2022).

### Toxicology of Algal Growth Rate Inhibition

To assess the acute effects, a 24–96 h period is the ideal time (Schuwirth, 2020). In order to evaluate temporary exposure toxicity of single and combined effects of PS-MPs and SDZ, algae were exposed to SDZ, PS-MPs, and combined SDZ with PS-MPs for 96 h. Since PPCPs were detected in a broad range of 0.1–50 mg l^–1^ in wastewater and *Chlamydomonas* was capable of removal of SDZ by photolysis (Xie et al., 2020), six concentrations of SDZ (5, 10, 20, 50, 100, and 200 mg l^–1^) and six concentrations of PS-MPs (5, 10, 20, 50, 100, and 200 mg l^–1^) were selected in this study and then the combination of fixed 50 mg l^–1^ PS-MPs and six concentrations of SDZ (5, 10, 20, 50, 100, and 200 mg l^–1^) to analysis their conjoint effects based on the results of individual toxicity experiments. *C. reinhardtii* cells were cultured in a 12-well plate (Thermo Fisher Scientific, Cat. No. 150628) and seeded in the concentration of 10^5^ cells ml^–1^; the cell concentration was counted using a hemocytometer for inhibition rate estimation.

It is known that *C. reinhardtii* generally reached stationary stage after 5–6 days of incubation. According to the results of 96 h inhibition tests, the microalgae were subjected to a 7-day toxicity test with 50 mg l^–1^ SDZ, 50 mg l^–1^ PS-MPs, and combined 50 mg l^–1^ SDZ with 50 mg l^–1^ PS-MPs. The microalgae were cultured in 250 ml flasks and kept the other culture conditions consistent with the previous conditions. After exposure, cell concentration and biomass were calculated. A 50-ml culture was collected in order to determine the concentration of extracellular secretory protein and the rest of the culture was centrifugated at 4,500 *g* and 20°C for 10 min to collect algal cells. The total protein was extracted to measure biochemical activities.

### Analysis of Chlorophyll Contents and Photosynthetic Activity Parameters

The contents of chlorophyll (Chl) were determined according to a modified method of the previous study (Zheng et al., 2017). The culture of algae (1 ml) was centrifuged at 12,000 rpm (Thermo Scientific Heraeus Pico 17 microcentrifuge) for 30 s at 20°C; then, the pellet was resuspended in 1 ml of 95% ethanol and incubated for 20 min at room temperature under shade condition and centrifuged again. The absorbance of the supernatant was measured at 630, 647, 664, and 750 nm using the Synergy Neo2 Plate Reader (BioTek, United States) and the contents of chlorophyll extract were calculated according to the following equations:


$$\mathrm{Chla}=[11.85\left(OD_{664}-OD_{750}\right)-1.54\left(OD_{647}-OD_{750}\right)-0.08\left(OD_{630}-OD_{750}\right)]$$

$$\mathrm{Chlb}=[-5.43\left(OD_{664}-OD_{750}\right)+21.03\left(OD_{647}-OD_{750}\right)-2.66\left(OD_{630}-OD_{750}\right)]$$

$$\mathrm{Chlc}=[-1.67\left(OD_{664}-OD_{750}\right)-7.6\left(OD_{647}-OD_{750}\right)+24.52\left(OD_{630}-OD_{750}\right)]$$

The parameters of photosynthetic activity of algae were measured using a pulse amplitude modulated (PAM) fluorometer water-PAM (Walz, Germany; (Zheng et al., 2017). Fv/Fm (Fv, variable fluorescence; Fm, maximum fluorescence) is the largest photochemical quantum yield of photosystem II (PSII), which reflects the quantum yield when all the PSII reaction centers are in an open state. Yield (YII) is the actual photochemical efficiency of PSII in light.

### Measurement of Antioxidant Activity of Enzyme

The activities of total antioxidant enzyme SOD and glutathione peroxidase (GSH-Px) were measured by using and following the recommended instructions of the colorimetric commercial kits (Cat. No. A001-3-2 and A005-1-2, respectively, Nanjing Jiancheng Bioengineering Research Institute). The activity of SOD was assayed with the reaction based on its inhibition on the scale of superoxide anion generated by xanthine and xanthine oxidase reaction system. A SOD unit was measured as the amount of enzyme that led to a half inhibition of the nitroblue tetrazolium reduction rate using the plate reader at 550 nm. GSH-Px was measured according to the manufacturer’s protocol. Based on the reaction ability of dithodinitrobenzoic acid with sulfhydryl compounds at 405 nm absorption peak in producing a relatively stable yellow color, GSH activity was measured. GSH-Px is preferably represented by catalyzed GSH reaction rate by measuring absorbance at 412 nm for 5 min. In this study, the activities of SOD and GSH-Px were expressed as units per milligram of protein (U mg^–1^). The concentration of protein was determined using a protein quantification kit (C503061-1250, Sangon Biotech, China).

### Confocal Laser Microscope and Scanning Electron Microscope Observation

After 96 h exposure, MPs and microalgae in culture were observed using a confocal laser scanning microscope (CLSM; Leica TCS SP8, Germany) and analyzed by image analysis system with HCX PL APO 40X 0.85 dry objective lens. Fluorescence images were recorded for MPs particles with excitation and emission wavelengths of 554 and 586 nm, respectively, and 649/670 nm for the determination of fluorescence for microalgal cells, respectively. MPs particles are shown in green color and microalgal cells are shown in red color. After 96 h of exposure under toxicity assay, the microalgal cells were collected by centrifugation (5,000 rpm, 10 min, and 20°C). Samples were initially fixed overnight at 4°C with 2.50% glutaraldehyde and then washed three times with phosphate-buffered saline (PBS; pH 7.4). Afterward, the samples were dehydrated for 15 min through a series of 30, 50, 70, 80, 90, 95, and 100% alcohol solutions. The dehydrated samples were placed in an oven at 40°C and dried for 24–48 h. After gold spraying, the samples were observed by scanning electron microscopy (SEM; Phenom Pro).

### Extracellular Secretory Protein and Sulfadiazine Removal Rate Measurement

The protein, which was extracted from extracellular polymeric substances (EPSs), was quantified and analyzed as follows: by 10,000 *g* centrifugation at 20°C for 15 min, supernatant solution was pipetted and filtered through a 0.45-μm glass fiber (GF) membrane (GF, 25 mm, mesh size 0.45 μm, Munktell). Colorimetric analysis of EPS protein content was carried out using the protein quantification kit (Sangon Biotech, China). The absorbance of the samples was read at 595 nm using the Synergy Neo2 Plate Reader (BioTek, United States) and protein concentration was calculated by comparison with the standard curve (Bellingeri et al., 2019).

The quantum yield is a critical factor that employs to quantify the efficiency of sulfonamides photodegradation reaction and an absorption peak at 254 nm indicates the photolysis products from SDZ degradation (Liu et al., 2018). Therefore, concentration of SDZ in algal culture was measured for antibiotics removal ratio assessment. A 50-ml of algal culture was centrifuged at 4,500 *g* and 20°C for 15 min; the supernatant was collected and SDZ concentration was measured at 254 nm using the plate reader and calculated by comparison with the standard curve.

### Statistical Analysis

Each experiment was performed in triplicate and graphs were plotted by the Prism 8 (GraphPad Software Incorporation). For statistical analysis, data were subjected to IBM SPSS version 25.0 (IBM SPSS) with the Shapiro–Wilk’s and Levene’s tests (*p* > 0.05) to confirm normal distribution and homoscedasticity, respectively. The differences among the treatments were analyzed by single-factor ANOVA and taking a level of *p* < 0.05 as significant to Duncan’s multiple range test. The difference and interactive effects between antibiotics and MPs were examined using the two-way ANOVA followed by the Student–Newman–Keuls (SNK) tests for multiple comparisons (with significant level of *p* < 0.05).

## Results and Discussion

### Inhibition Effect of Polystyrene-Microplastics and Sulfadiazine on Microalgae Growth

**FIGURE 1:**
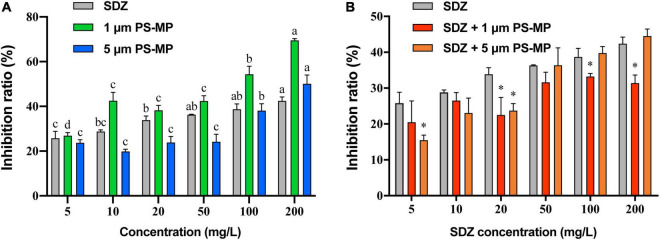
The average growth inhibition ratio of microalgae exposed 96 h to sulfadiazine (SDZ) and polystyrene-microplastics (PS-MPs) in the different groups. **(A)** Single treatment of SDZ and MPs and **(B)** 50 mg l^–1^ of different particle size MPs + SDZ in comparison with SDZ only. Values are the mean ± SEM of three replicates. Significant differences (*p* < 0.05) are denoted with different letters **(A)** and an asterisk for MPs + SDZ vs. SDZ only **(B)**.

The toxicity of SDZ and PS-MPs on the growth of microalgae was evaluated by the growth inhibition effect. The results showed that the exposure of PS-MPs and SDZ treatment alone inhibited the growth of algal cells and the inhibition effect was gradually increased at higher concentration. For instance, at the highest tested concentration of 200 mg l^–1^, the PS-MPs alone inhibited the algal growth rate of 70.41 and 50.41% by using 1 and 5 μm, respectively, while SDZ alone inhibited only 42.40% (Figure 1A). It indicates that individual PS-MPs exposure inhibited the growth of *C. reinhardtii* cells. Under the same concentration, for different particle size of MPs, smaller size of particle had a higher inhibition rate of microalgae growth and the inhibition effect was also enhanced with an increasing concentration for 1 and 5 μm PS-MPs. This is similar to the findings, which have been already reported in other organisms (e.g., rotifers, shrimps, fungus), also showed that MPs with smaller sizes of particles were more toxic than those with larger sizes of particles (Varó et al., 2019; Wang et al., 2019a,b). There may probably three main reasons, which are associated to this phenomenon: first, smaller diameter of MPs may be able to enter the cell and affect the microalgae directly (Chen et al., 2020b). Besides, smaller diameter of MPs is more likely to adsorb to the surface of the algal cell and ultimately affect the uptake of external nutrients (Bhattacharya et al., 2010). Finally, the presence of MPs can have a shading effect on microalgae, thereby affecting the photosynthesis of algal cells and causing toxicity. Under the same concentration of MPs, the number of small size of MPs particles is much higher than that of large size of MPs particles, leading to a lower light transmission rate for small size MPs cultures, thus affecting algal cell growth (Zhang et al., 2017). However, microalgal cells are highly resistant to SDZ antibiotics and the inhibition rate was remained below 50% at SDZ concentrations up to 200 mg l^–1^, suggesting that algal cells can remove antibiotics from the environment. It was shown that microalgae could remove antibiotics by photolysis and biodegradation (Xie et al., 2020).

As the analysis showed that there was no significant difference in inhibition rate of single treatment PS-MPs concentration between 10 and 50 mg l^–1^, the result of algal cell inhibition rates at fixed concentrations (50 mg l^–1^) of PS-MPs combined with different concentrations of antibiotics was subsequently analyzed (Figure 1B). With contents of SDZ below 50 mg l^–1^, the joint inhibition rate seems consistently lower than SDZ presence alone. When concentration of SDZ was above 50 mg l^–1^, the rate of inhibition of PS-MPs with smaller sizes of particle was lower than that of larger sizes of particle. For instance, the combined inhibition of SDZ with 1 μm PS-MPs decreased by 5.43% (*p* < 0.05) at SDZ concentration of 100 mg l^–1^ compared to SDZ alone, while this value decreased by 11.0% (*p* < 0.05) at SDZ concentration of 200 mg l^–1^. In contrast, there was no significant difference between SDZ alone and in combination with SDZ with 5 μm PS-MPs at SDZ concentrations of 100 and 200 mg l^–1^. This evidence is suggesting that the presence of MPs may reduce the inhibition of algal growth by excessive antibiotics. This effect is notable for MPs with smaller sizes of particle. The two-way ANOVA results indicated significant effects of MPs and SDZ for growth inhibition of *C. reinhardtii* and the interaction between SDZ and 5 μm PS-MPs (Supplementary Table 1). MPs have a strong adsorption capacity for heavy metals, organic pollutants, etc. (Barboza et al., 2018), which could reduce the concentrations of antibiotics in the medium. At the same concentration and with a larger specific surface area, small size of MPs is more powerful in adsorption and, thus, more effective in reducing the inhibition rate (Sjollema et al., 2016). In the control group exposed only to SDZ, the toxicity of SDZ may be reflected in an increased oxidative stress and chlorophyll inhibition in algal cells, thereby reducing photosynthetic efficiency and affecting algal growth. The secretion of EPS by algal cells makes it easier for MPs of small sizes to accumulate around algal cells, potentially forming a barrier and reducing SDZ exposure. Consequently, the two particle sizes of MPs interact differently with SDZ. As MPs compete with SDZ for toxicity, the combined action of SDZ and MPs reduced the rate of growth inhibition of *C. reinhardtii*.

**FIGURE 2:**
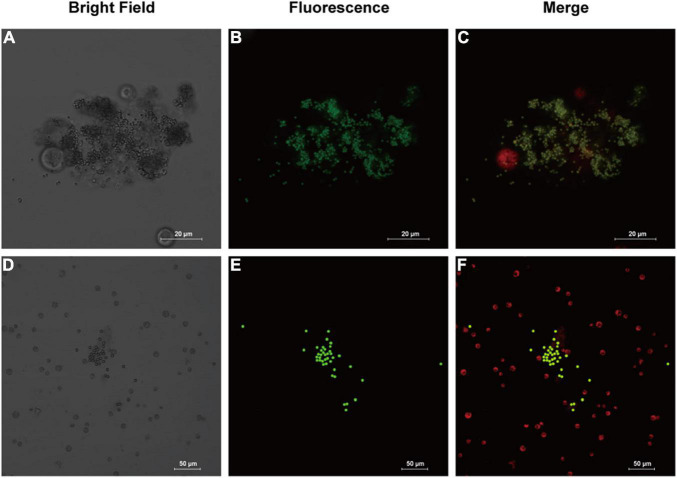
Images of fluorescently labeled PS-MPs in microalgal culture (96 h). **(A–C)**: 1 μm MPs in culture with *Chlamydomonas reinhardtii* (*C. reinhardtii*), bright field **(A)** the fluorescence of MPs distribution **(B)** and merged fluorescence of microalgae (red) and MPs (green) **(C)**. **(D–F)**: 5 μm MPs in culture with *C. reinhardtii*, bright field **(D)** the fluorescence of MPs **(E)** and merged fluorescence **(F)**.

Analysis of morphology of microalgal cell can provide more clues to the health status of microalgae, as well as indications of the effects of combining MPs and antibiotics on microalgae. According to the confocal microscopic image (Figure 2), it was observed that the MPs were not uniformly distributed and aggregation occurred. The aggregation was evident in microalgae treated with 1 μm PS-MPs, which was likely due to the fact that the algal cells secreted a large amount of extracellular organic matter, which caused the MPs to stick, leading to the deposition and aggregation of MPs at the bottom. Therefore, the concentration of MPs in the medium is decreased and may reduce the toxicity of MPs to algal cells. Stimulation by different excitation light can discriminate between the fluorescence of chlorophyll and MPs; it was found that most of the algal cells did not directly contact with MPs and only a small amount of MPs would be contacted with the surface of algal cells, but no MPs were found to enter the algal cells, which may indicate that the inhibitory effect on algal cells is not caused by internalization of the small-diameter MPs, but possibly by influencing the external environment such as nutrient uptake and light radiation to algal cells. However, another study provided evidence that 1–2 μm MPs could enter cells of two species of microalgae at extremely low rates, but the detailed mechanisms remain undiscovered (Yang et al., 2020).

**FIGURE 3:**
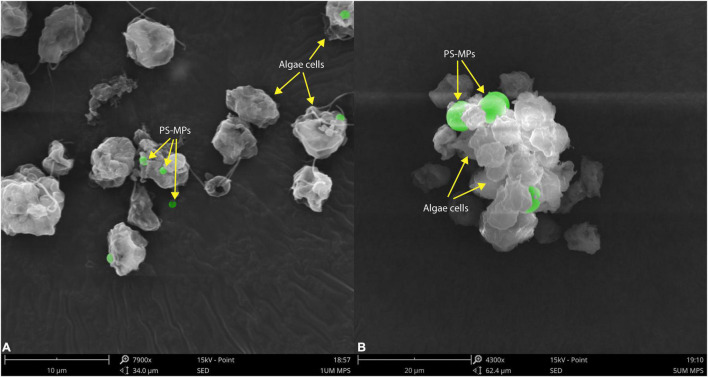
Scanning electron microscopic images of *C. reinhardtii* exposed to different particle size of PS-MPs **(A)** 1 μm MPs; **(B)** 5 μm MPs. The identified MPs are green colored.

The morphological characteristics of *C. reinhardtii* under different treatments were observed by scanning electron microscopy. PS-MPs at the concentration of 1 μm could attach to the surface of *C. reinhardtii* (Figure 3A) and PS-MPs at the concentration of 5 μm could not attach to the surface of microalgae due to their large size, but would aggregate with microalgae to form a cluster (Figure 3B). The aggregation of MPs and microalgae may cause physical damage to the algae, such as changes in structure of cell wall (Wang et al., 2016), furthermore inhibiting the growth of the algae. In addition, this aggregation can increase the sedimentation coefficient of microalgae, consequently leading to settling of microalgae and aggregation at the bottom of the flask in the later stages of culture.

### Antioxidant Enzymes and Photosynthesis Performance Under Microplastics and Sulfadiazine Toxicity

**FIGURE 4:**
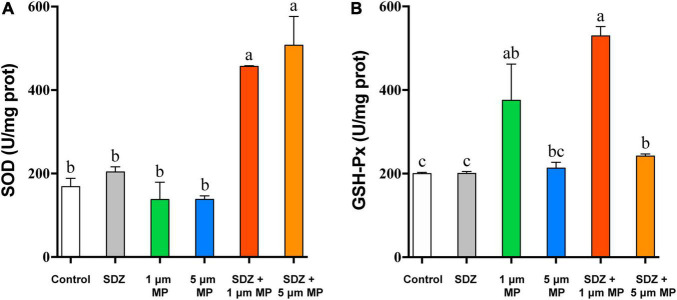
The superoxide dismutase (SOD) **(A)** and glutathione peroxidase (GSH-Px) **(B)** enzyme activities of *C. reinhardtii* exposed to PS-MPs (1 and 5 μm) and/or SDZ (50 mg l^–1^). Values are means ± SEM of three replicates and significant differences (*p* < 0.05) are denoted with different letters.

When the external environment is unfavorable to the growth of microalgae, it causes conducive changes to the growth of algae for their survival and this process is mainly regulated by the activity of some enzymes in algal cells (Li et al., 2020a). The existence of MPs and antibiotics in the external environment is involved in affecting the enzymatic activity of algal cells, but to significantly different extents. Comparison of enzyme activity (Figure 4) showed that the intracellular GSH-Px activity was basically same as the control under the condition of individual treatment of SDZ at the concentration of 50 mg l^–1^, which indicated that antibiotics at this concentration did not affect the intracellular GSH-Px enzyme activity, but when MPs were present in the medium, the intracellular GSH-Px activity was significantly increased in all the cases. The enzyme activity was peaked at the diameter of 1 μm PS-MPs and 50 mg l^–1^ SDZ in combined treatment (Figure 4B), suggesting that particle size of MPs affects the growth of algal cell, which is consistent with other previously described studies (Wu et al., 2021). The SOD enzyme activity assay revealed that the results were consistent with GSH-Px, but the highest SOD activity was observed in the presence of 5 μm PS-MPs and SDZ (Figure 4A). The two-way ANOVAs revealed significant main effects of MPs, but not SDZ for GSH-Px activity and the effects of MPs on GSH-Px were independent of SDZ. The SOD activity was significantly responsive to MPs and SDZ and the interactions between MPs and SDZ (Supplementary Table 2). Studies have suggested that stress from external environmental factors, including heavy metals, organic acids, and salt stress, can induce significant production of ROS inside the plant cells (Cheng et al., 2017). It was found in previous study of the toxicity of aging microplastic polyvinyl chloride and copper to microalgae *Chlorella vulgaris* that both the mPVC (10 mg l^–1^) and copper (0.5 mg l^–1^) caused severe cellular damage and increased the concentrations of intracellular SOD and malondialdehyde (MDA; Fu et al., 2019). Excess ROS could damage the cell membrane system of the organism and eventually inhibited their growth. Mitochondria are a major source of free radicals and MPs can interact with their outer membranes (reducing mitochondrial membrane potential) or indirectly affect their function, thus resulting in ROS production. As frontline defense enzymes that directly eliminate ROS, both the SOD and GSH-Px are potentially effective markers of early oxidative damage induced by MPs and antibiotics. The SOD catalyzes the dismutation of O_2_^–^ to molecular oxygen and hydrogen peroxide (H_2_O_2_), which is subsequently removed by catalase and GSH-Px. These enzymes catalyze the reduction of H_2_O_2_ to harmless products and the intracellular antioxidative system scavenges excess oxygen radicals, leading to avoiding or reducing oxidative damage (Cheng et al., 2017; Prokić et al., 2019).

**FIGURE 5:**
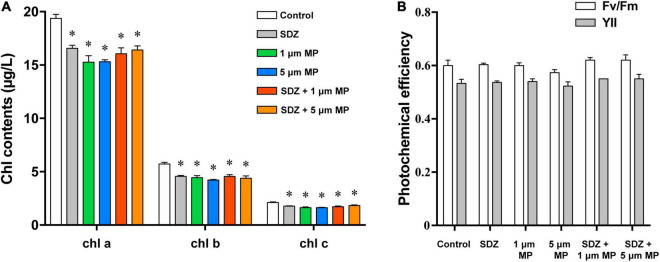
The chlorophyll contents **(A)** and photochemical efficiency **(B)** of *C. reinhardtii* exposed to PS-MPs (1 and 5 μm) and/or SDZ (50 mg l^–1^). Data are shown as means ± SEM of three replicates and an asterisk note for significant difference between treatments and the control group (*p* < 0.05).

The chlorophyll content of microalgae in the presence of both the MPs and SDZ was measured in this study and the results (Figure 5A) showed a higher chlorophyll content in the control group and both the presence of SDZ and MPs alone and in combination resulted in a significant reduction of chlorophyll a, b, and c in algal cells, with inhibition rates of roughly 15–23%. This is following the results of other studies, except for the difference in the inhibition effect, e.g., it was found that the pigment content within microalgae was significantly reduced after 48 h of MPs exposure, with a maximum inhibition rate of 62.86% (Yang et al., 2021). This may be related to the tolerance of different species of microalgae and the material of MPs (Zhu et al., 2019). It appears that *C. vulgaris* was less sensitive to SDZ and the chlorophyll a content decreased significantly in the 10 and 270 mg l^–1^ groups following 7-day exposure, but only slightly reduced in the 30 and 90 mg l^–1^ groups (Chen et al., 2020a). In contrast, *C. reinhardtii* was more sensitive in this experiment and the chlorophyll contents were all significantly decreased after treatment. The two-way ANOVAs revealed that chlorophyll a and c contents were not significantly affected by SDZ, but all the chlorophyll contents were significantly responsive to MPs exposure and their interaction between MPs and SDZ (Supplementary Table 3). Chlorophyll fluorescence is sensitive parameter to environmental stress; the decrease in contents of chlorophyll may be related to the accumulation of intracellular ROS that disrupts the cell structure and hinders the synthesis of chlorophyll. In addition, it may be due to the high adsorption capacity of MPs, which adsorb on microalgae to form aggregates during the culture process, rendering some algae inactive (Lagarde et al., 2016). We have observed in this study that there was no significant difference in the contents of chlorophyll in combined treatments (MPs and SDZ) compared to their individual treatments, suggesting that the combined effect of MPs and SDZ on chlorophyll in *C. reinhardtii* is consistent with their presence alone.

Photosynthetic efficiency of microalgae was measured using the PHYTO-PAM fluorometer to examine whether MPs and SDZ affect the photosynthesis of microalgae. Fv/Fm and YII represent the ability of PSII to absorb photon energy and use it for photochemistry under dark-adapted and light-adapted conditions, respectively, when damage or inhibition of protein complexes in the photosystem and reduced viability of algal cells may result in the declined photosynthetic capacity of microalgal cells (Schwab et al., 2011). The results, which are shown in Figure 5B, indicated the photosynthetic efficiency of microalgae, despite a slight variance was not statistically different, suggesting that the photosynthetic efficiency of microalgae was not affected by PS-MPs and antibiotics. According to the two-way ANOVA analysis, photosynthetic efficiency was significantly affected by SDZ, but not significantly responsive to MPs and interaction between MPs and SDZ (Supplementary Table 4). In a previous study, the results were similar, which indicated that MPs (25 mg l^–1^, sizes 0.05, 0.5, and 6 μm) did not affect the photosynthesis of various microalgae, including diatoms, *Dunaliella salina*, and freshwater *Chlorella* (Sjollema et al., 2016). However, another study revealed that a unicellular flagellate alga *Karenia mikimotoi* showed decreasing trend in photosynthesis about 25.3 and 17.1% in YII and Fv/Fm, respectively, when exposed it under 0–100 mg l^–1^ 1.0 μm PVC-MPs (Zhao et al., 2019). We have inferred from these findings that the sensitivity of different microalgae to MPs differs and may also be associated with the food web and phytoplankton community in aquatic ecosystems.

### Extracellular Secretory Proteins and Removal of Sulfadiazine Antibiotics by Microalgae

**FIGURE 6:**
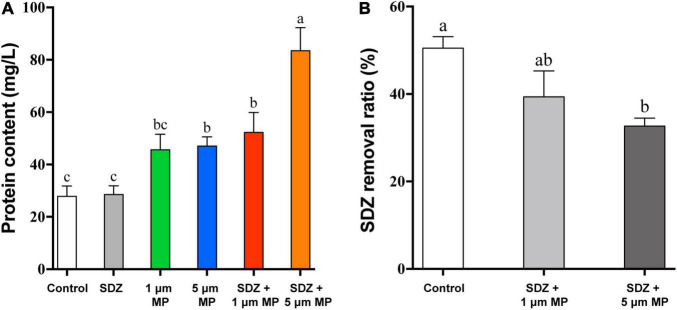
The extracellular cellular protein content of *C. reinhardtii* exposed to PS-MPs (1 and 5 μm) and/or SDZ (50 mg l^–1^) **(A)** and SDZ removal ratio with different particle sizes PS-MPs coexistence **(B)**. Values are means ± SEM of three replicates and significant differences (*p* < 0.05) are denoted with different letters.

In response to diverse environmental stresses, microalgal cells secrete the natural EPS, which provides functions as a barrier to prevent the entry of toxic substances into the cell, triggers the induction of protective mechanisms in response to unfavorable growth conditions from the external environment, and then secretes extracellular proteins to protect the cell (Bellingeri et al., 2019). Thus, the presence of MPs may stimulate the secretion of EPS by microalgae, leading to high protein contents in the culture medium. To test this hypothesis, extracellular secretory proteins were measured after 7 days of PS-MPs and SDZ treatment and the results (Figure 6A) showed that individual treatment of SDZ did not increase the amount of secreted extracellular protein. MPs alone and in combination with SDZ increased the amount of extracellular protein secreted by microalgal cells. The amount of extracellular protein, which is secreted by microalgal cells, reached at its maximum stage upon combined treatments of 5 μm PS-MPs and antibiotics. The results of two-way ANOVA showed that SDZ and MPs had a significant effect on EPS production and the interaction analysis of SDZ and MPS revealed a correlation between them (Supplementary Table 5). The variation in the quantity and composition of EPS secretion can be influenced by environmental conditions and the discrepancies in the combined effects of MPs and antibiotics of different sizes of particle may be the primary factor, which are contributing to the amount of secreted extracellular proteins (Adeleye and Keller, 2016). After 9 h of 2 μm PS-MPs exposure, the bound and soluble EPS of microalgae *Scenedesmus abundans* significantly increased (Cheng and Wang, 2022). In another study, *C. reinhardtii* was exposed to 50 mg l^–1^ of PS-MPs (300–600 nm) for 6 days and EPS decreased slightly compared to the control group, but remained much higher than the level at the start of this study (Li et al., 2020b). Therefore, stimulus of MPs to microalgae may show different features (e.g., biomass, growth inhibition, and quantity of EPS) depending on species, size, and treatment modality.

The most antibiotics are difficult to biodegrade due to their pharmacological stability (Martin-Laurent et al., 2019) or resistance (Li et al., 2020a), causing the residues of antibiotics in the environment, which are notable for their harmful effects on the ecology and human health. In previous studies, microorganisms were isolated, which have potential capacity to biodegrade the effect of antibiotic, such as SDZ. It was noticed that strain of *Arthrobacter* D2 degrades the effects of SDZ more than 50% at an initial concentration of 50 mg l^–1^ (Deng et al., 2016). The studies have been demonstrated that microalgae have great prospective to eliminate antibiotics from the aqueous environment through multiple differential mechanisms. The most effective pathways, which are involving in the removal of organic pollutants by microalgae, are performed through enzymatic reactions, mainly including oxidation and hydrolysis (Foflonker et al., 2016; Xiong et al., 2017). There are also studies showing biosorption of antibiotics by microalgae cells (Angulo et al., 2018), while compounds with the cationic groups tend to adsorb on the surface of microalgae through electrostatic interactions (Xiong et al., 2019). In addition, abiotic reactions, including hydrolysis and photolysis, also contribute to the removal of antibiotics (Norvill et al., 2017; Song et al., 2019). A study revealed that removal efficiency of SDZ by *Chlamydomonas* spp. exceeded 50% at an initial concentration of 10 mg l^–1^. In PPCPs removal, adsorption played an essential role, ciprofloxacin and SDZ are mainly adsorbed on EPS by binding with the carbonyl and amine groups in tryptophan protein-like substances, and EPS on the microalgae is limited, making it easier for PPCPs to pass through and diffuse into the cells for biodegradation. However, due to the negative zeta potential, SDZ has lower adsorption efficiency with EPS (Xie et al., 2020). To study whether MPs affect the bioremoval of SDZ by microalgae, after 7 days of culture, SDZ concentration in the medium was measured. The removal rate of SDZ reached up to 51% in microalgae medium, which are treated with only 50 mg l^–1^ SDZ (control), while the removal rate decreased by 11 and 18% in the medium supplemented with 1 μm PS-MPs and 5 μm PS-MPs, respectively (Figure 6B). The increase of EPS, particularly in the 5 μm MPs group and the relatively low adsorption of SDZ onto microalgal EPS, could affect algal internalization of SDZ, which in conjunction with the cytotoxicity of PS-MPs may reduce SDZ removal efficiency. The results showed that bioremoval of SDZ by microalgae was inhibited in the presence of PS-MPs; this is consistent with a recent study showing that levofloxacin removal by *C. vulgaris* was significantly inhibited when PS-MPs co-exposed (Wu et al., 2022); even though the antibiotic species and MPs have different effects on their degradation mechanisms, understanding the scavenging of antibiotics from the environment by microalgae and the mechanisms is important.

## Conclusion

In this study, we examined the single and combined effects of PS-MPs and SDZ on freshwater alga *C. reinhardtii*. SDZ exposure and the attachment of MPs to microalgae increased antioxidant enzyme activity, resulting in an increase in extracellular secretory proteins and a decrease in chlorophyll content. This was accompanied by a reduction in the growth rate of microalgae and noticeable aggregation of MPs. Exposure toxicity increased with the concentration and interaction of PS-MPs and SDZ, but different particle sizes and their interactions with SDZ had differential effects and when co-exposed with 5 μm PS-MPs, SDZ removal rates by algae were compromised. Therefore, considering the increasing trend of global antibiotics and microplastics production in response to the demand of the human population growing rapidly and the fundamental role of microalgae as primary producers in aquatic ecosystems, more studies on the combined effects of microplastics and emerging contaminants are required.

## Data Availability Statement

The original contributions presented in the study are included in the article/Supplementary Material, further inquiries can be directed to the corresponding author/s.

## Author Contributions

ZL, YZ, and ZH conceived and designed the experiments. ZL and LL performed the experiments. ZL, SD, and FH analyzed the data. ZL and YZ contributed reagents, materials, analysis tools, and wrote the manuscript. All authors contributed to the article and approved the submitted version.

## Conflict of Interest

The authors declare that the research was conducted in the absence of any commercial or financial relationships that could be construed as a potential conflict of interest.

## Publisher’s Note

All claims expressed in this article are solely those of the authors and do not necessarily represent those of their affiliated organizations, or those of the publisher, the editors and the reviewers. Any product that may be evaluated in this article, or claim that may be made by its manufacturer, is not guaranteed or endorsed by the publisher.

## References

[B1] Adeleye A. S. Keller A. A. (2016). Interactions between algal extracellular polymeric substances and commercial TiO2 nanoparticles in aqueous media. *Environ. Sci. Technol.* 50 12258–12265. 10.1021/acs.est.6b03684 27766831

[B2] Angulo E. Bula L. Mercado I. Montano A. Cubillan N. (2018). Bioremediation of cephalexin with non-living *Chlorella* sp., biomass after lipid extraction. *Bioresour. Technol.* 257 17–22. 10.1016/j.biortech.2018.02.079 29477662

[B3] Barboza L. G. A. Vieira L. R. Branco V. Figueiredo N. Carvalho F. Carvalho C. (2018). Microplastics cause neurotoxicity, oxidative damage and energy-related changes and interact with the bioaccumulation of mercury in the European seabass, *Dicentrarchus labrax* (Linnaeus, 1758). *Aquat. Toxicol.* 195 49–57. 10.1016/j.aquatox.2017.12.008 29287173

[B4] Beiras R. Verdejo E. Campoy-López P. Vidal-Liñán L. (2021). Aquatic toxicity of chemically defined microplastics can be explained by functional additives. *J. Hazard. Mater.* 406:124338. 10.1016/j.jhazmat.2020.124338 33525131

[B5] Bellingeri A. Bergami E. Grassi G. Faleri C. Redondo-Hasselerharm P. Koelmans A. A. (2019). Combined effects of nanoplastics and copper on the freshwater alga *Raphidocelis subcapitata*. *Aquat. Toxicol.* 210 179–187. 10.1016/j.aquatox.2019.02.022 30870664

[B6] Bhattacharya P. Lin S. Turner J. Ke P. (2010). Physical adsorption of charged plastic nanoparticles affects algal photosynthesis. *J. Phys. Chem. C* 114:16556. 10.1021/jp1054759

[B7] Brandon J. Goldstein M. Ohman M. D. (2016). Long-term aging and degradation of microplastic particles: comparing *in situ* oceanic and experimental weathering patterns. *Mar. Pollut. Bull.* 110 299–308. 10.1016/j.marpolbul.2016.06.048 27344287

[B8] Chae Y. Kim D. An Y.-J. (2019). Effects of micro-sized polyethylene spheres on the marine microalga *Dunaliella salina*: focusing on the algal cell to plastic particle size ratio. *Aquat. Toxicol.* 216:105296. 10.1016/j.aquatox.2019.105296 31541944

[B9] Chaturvedi P. Shukla P. Giri B. S. Chowdhary P. Chandra R. Gupta P. (2021). Prevalence and hazardous impact of pharmaceutical and personal care products and antibiotics in environment: a review on emerging contaminants. *Environ. Res.* 194:110664. 10.1016/j.envres.2020.110664 33400949

[B10] Chen S. Wang L. Feng W. Yuan M. Li J. Xu H. (2020a). Sulfonamides-induced oxidative stress in freshwater microalga *Chlorella vulgaris*: evaluation of growth, photosynthesis, antioxidants, ultrastructure, and nucleic acids. *Sci. Rep.* 10:8243. 10.1038/s41598-020-65219-2 32427937 PMC7237458

[B11] Chen Y. Ling Y. Li X. Hu J. Cao C. He D. (2020b). Size-dependent cellular internalization and effects of polystyrene microplastics in microalgae *P. helgolandica* var. *tsingtaoensis* and *S. quadricauda*. *J. Hazard. Mater.* 399:123092. 10.1016/j.jhazmat.2020.123092 32531675

[B12] Cheng J. Ye Q. Yang Z. Yang W. Zhou J. Cen K. (2017). Microstructure and antioxidative capacity of the microalgae mutant *Chlorella* PY-ZU1 during tilmicosin removal from wastewater under 15% CO2. *J. Hazard. Mater.* 324 414–419. 10.1016/j.jhazmat.2016.11.006 27829514

[B13] Cheng Y.-R. Wang H.-Y. (2022). Highly effective removal of microplastics by microalgae *Scenedesmus abundans*. *Chem. Eng. J.* 435:135079. 10.1016/j.cej.2022.135079

[B14] Coyle R. Hardiman G. Driscoll K. O. (2020). Microplastics in the marine environment: a review of their sources, distribution processes, uptake and exchange in ecosystems. *Case Stud. Chem. Environ. Eng.* 2:100010. 10.1016/j.cscee.2020.100010

[B15] Deng Y. Mao Y. Li B. Yang C. Zhang T. (2016). Aerobic degradation of sulfadiazine by *Arthrobacter* spp.: kinetics, pathways, and genomic characterization. *Environ. Sci. Technol.* 50, 9566–9575. 10.1021/acs.est.6b02231 27477918

[B16] Elizalde-Velázquez G. A. Gómez-Oliván L. M. (2021). Microplastics in aquatic environments: a review on occurrence, distribution, toxic effects, and implications for human health. *Sci. Total Environ.* 780:146551. 10.1016/j.scitotenv.2021.146551 33773347

[B17] Foflonker F. Ananyev G. Qiu H. Morrison A. Palenik B. Dismukes G. C. (2016). The unexpected extremophile: tolerance to fluctuating salinity in the green alga Picochlorum. *Algal Res.* 16 465–472. 10.1016/j.algal.2016.04.003

[B18] Fu D. D. Zhang Q. J. Fan Z. Q. Qi H. Y. Wang Z. Z. Peng L. C. (2019). Aged microplastics polyvinyl chloride interact with copper and cause oxidative stress towards microalgae *Chlorella vulgaris*. *Aquat. Toxicol.* 216:105319. 10.1016/j.aquatox.2019.105319 31586885

[B19] Holmes L. A. Turner A. Thompson R. C. (2012). Adsorption of trace metals to plastic resin pellets in the marine environment. *Environ. Pollut.* 160 42–48. 10.1016/j.envpol.2011.08.052 22035924

[B20] Huang G. Zhao D. Lan C. Wu B. Li X. Lou S. (2022). Glucose-assisted trophic conversion of *Chlamydomonas reinhardtii* by expression of glucose transporter GLUT1. *Algal Res.* 62:102626. 10.1016/j.algal.2021.102626

[B21] Lagarde F. Olivier O. Zanella M. Daniel P. Hiard S. Caruso A. (2016). Microplastic interactions with freshwater microalgae: hetero-aggregation and changes in plastic density appear strongly dependent on polymer type. *Environ. Pollut.* 215 331–339. 10.1016/j.envpol.2016.05.006 27236494

[B22] Li H. Wei L. Lu J. (2020a). Algae-induced photodegradation of antibiotics: a review. *Environ. Pollut.* 272:115589. 10.1016/j.envpol.2020.115589 33234380

[B23] Li S. Wang P. Zhang C. Zhou X. Yin Z. Hu T. (2020b). Influence of polystyrene microplastics on the growth, photosynthetic efficiency and aggregation of freshwater microalgae *Chlamydomonas reinhardtii*. *Sci. Total Environ.* 714:136767. 10.1016/j.scitotenv.2020.136767 31981864

[B24] Liu X. Liu Y. Lu S. Guo W. Xi B. (2018). Performance and mechanism into TiO2/Zeolite composites for sulfadiazine adsorption and photodegradation. *Chem. Eng. J.* 350 131–147. 10.1016/j.cej.2018.05.141

[B25] Machado M. D. Soares E. V. (2019). Impact of erythromycin on a non-target organism: cellular effects on the freshwater microalga *Pseudokirchneriella subcapitata*. *Aquat. Toxicol.* 208 179–186. 10.1016/j.aquatox.2019.01.014 30682620

[B26] Martin-Laurent F. Topp E. Billet L. Batisson I. Malandain C. Besse-Hoggan P. (2019). Environmental risk assessment of antibiotics in agroecosystems: ecotoxicological effects on aquatic microbial communities and dissemination of antimicrobial resistances and antibiotic biodegradation potential along the soil-water continuum. *Environ. Sci. Pollut. Res.* 26 18930–18937. 10.1007/s11356-019-05122-0 31055743

[B27] Nava V. Leoni B. (2021). A critical review of interactions between microplastics, microalgae and aquatic ecosystem function. *Water Res.* 188:116476. 10.1016/j.watres.2020.116476 33038716

[B28] Norvill Z. N. Toledo-Cervantes A. Blanco S. Shilton A. Guieysse B. Muñoz R. (2017). Photodegradation and sorption govern tetracycline removal during wastewater treatment in algal ponds. *Bioresour. Technol.* 232 35–43. 10.1016/j.biortech.2017.02.011 28214443

[B29] Prata J. C. Lavorante B. R. B. O. Montenegro M. C. B. S. M. Guilhermino L. (2018). Influence of microplastics on the toxicity of the pharmaceuticals procainamide and doxycycline on the marine microalgae *Tetraselmis chuii*. *Aquat. Toxicol.* 197 143–152. 10.1016/j.aquatox.2018.02.015 29494946

[B30] Prokić M. D. Radovanović T. B. Gavrić J. P. Faggio C. (2019). Ecotoxicological effects of microplastics: examination of biomarkers, current state and future perspectives. *TrAC Trends Anal. Chem.* 111 37–46. 10.1016/j.trac.2018.12.001

[B31] Rath S. Fostier A. H. Pereira L. A. Dioniso A. C. de Oliveira Ferreira F. Doretto K. M. (2019). Sorption behaviors of antimicrobial and antiparasitic veterinary drugs on subtropical soils. *Chemosphere* 214 111–122. 10.1016/j.chemosphere.2018.09.083 30261417

[B32] Schuwirth N. (2020). Towards an integrated surface water quality assessment: aggregation over multiple pollutants and time. *Water Res.* 186:116330. 10.1016/j.watres.2020.116330 32911267

[B33] Schwab F. Bucheli T. D. Lukhele L. P. Magrez A. Nowack B. Sigg L. (2011). Are carbon nanotube effects on green algae caused by shading and agglomeration? *Environ. Sci. Technol.* 45 6136–6144. 10.1021/es200506b 21702508

[B34] Sjollema S. B. Redondo-Hasselerharm P. Leslie H. A. Kraak M. H. S. Vethaak A. D. (2016). Do plastic particles affect microalgal photosynthesis and growth? *Aquat. Toxicol.* 170 259–261. 10.1016/j.aquatox.2015.12.002 26675372

[B35] Song C. Wei Y. Qiu Y. Qi Y. Li Y. Kitamura Y. (2019). Biodegradability and mechanism of florfenicol *via Chlorella* sp. UTEX1602 and L38: experimental study. *Bioresour. Technol.* 272 529–534. 10.1016/j.biortech.2018.10.080 30391846

[B36] Sun Q. Ren S. Y. Ni H. G. (2020). Incidence of microplastics in personal care products: an appreciable part of plastic pollution. *Sci. Total Environ.* 742:140218. 10.1016/j.scitotenv.2020.140218 32629242

[B37] Varó I. Perini A. Torreblanca A. Garcia Y. Bergami E. Vannuccini M. L. (2019). Time-dependent effects of polystyrene nanoparticles in brine shrimp *Artemia franciscana* at physiological, biochemical and molecular levels. *Sci. Total Environ.* 675 570–580. 10.1016/j.scitotenv.2019.04.157 31030162

[B38] Wang J. Liu X. Dai Y. Ren J. Li Y. Wang X. (2020a). Effects of co-loading of polyethylene microplastics and ciprofloxacin on the antibiotic degradation efficiency and microbial community structure in soil. *Sci. Total Environ.* 741:140463. 10.1016/j.scitotenv.2020.140463 32886986

[B39] Wang Z. Gao J. Li D. Dai H. Zhao Y. (2020b). Co-occurrence of microplastics and triclosan inhibited nitrification function and enriched antibiotic resistance genes in nitrifying sludge. *J. Hazard. Mater.* 399:123049. 10.1016/j.jhazmat.2020.123049 32526436

[B40] Wang Y. Mao Z. Zhang M. Ding G. Sun J. Du M. (2019a). The uptake and elimination of polystyrene microplastics by the brine shrimp, *Artemia parthenogenetica*, and its impact on its feeding behavior and intestinal histology. *Chemosphere* 234 123–131. 10.1016/j.chemosphere.2019.05.267 31207418

[B41] Wang Y. Zhang D. Zhang M. Mu J. Ding G. Mao Z. (2019b). Effects of ingested polystyrene microplastics on brine shrimp, *Artemia parthenogenetica*. *Environ. Pollut.* 244 715–722. 10.1016/j.envpol.2018.10.024 30384077

[B42] Wang Y. Zhu X. Lao Y. Lv X. Tao Y. Huang B. (2016). TiO2 nanoparticles in the marine environment: physical effects responsible for the toxicity on algae *Phaeodactylum tricornutum*. *Sci. Total Environ.* 565 818–826. 10.1016/j.scitotenv.2016.03.164 27060054

[B43] Wright S. L. Thompson R. C. Galloway T. S. (2013). The physical impacts of microplastics on marine organisms: a review. *Environ. Pollut.* 178 483–492. 10.1016/j.envpol.2013.02.031 23545014

[B44] Wu D. Wang T. Wang J. Jiang L. Yin Y. Guo H. (2021). Size-dependent toxic effects of polystyrene microplastic exposure on *Microcystis aeruginosa* growth and microcystin production. *Sci. Total Environ.* 761:143265. 10.1016/j.scitotenv.2020.143265 33257060

[B45] Wu X. Wu H. Zhang A. Sekou K. Li Z. Ye J. (2022). Influence of polystyrene microplastics on levofloxacin removal by microalgae from freshwater aquaculture wastewater. *J. Environ. Manag.* 301:113865. 10.1016/j.jenvman.2021.113865 34597951

[B46] Xie P. Chen C. Zhang C. Su G. Ren N. Ho S.-H. (2020). Revealing the role of adsorption in ciprofloxacin and sulfadiazine elimination routes in microalgae. *Water Res.* 172:115475. 10.1016/j.watres.2020.115475 31972413

[B47] Xiong J.-Q. Govindwar S. Kurade M. B. Paeng K.-J. Roh H.-S. Khan M. A. (2019). Toxicity of sulfamethazine and sulfamethoxazole and their removal by a green microalga, *Scenedesmus obliquus*. *Chemosphere* 218 551–558. 10.1016/j.chemosphere.2018.11.146 30500716

[B48] Xiong J.-Q. Kurade M. B. Jeon B.-H. (2017). Ecotoxicological effects of enrofloxacin and its removal by monoculture of microalgal species and their consortium. *Environ. Pollut.* 226 486–493. 10.1016/j.envpol.2017.04.044 28449968

[B49] Yang S. Li Y. De Boevre M. De Saeger S. Zhou J. Li Y. (2020). Toxicokinetics of α-zearalenol and its masked form in rats and the comparative biotransformation in liver microsomes from different livestock and humans. *J. Hazard. Mater.* 393:121403. 10.1016/j.jhazmat.2019.121403 32143155

[B50] Yang W. Gao P. Li H. Huang J. Zhang Y. Ding H. (2021). Mechanism of the inhibition and detoxification effects of the interaction between nanoplastics and microalgae *Chlorella pyrenoidosa*. *Sci. Total Environ.* 783:146919. 10.1016/j.scitotenv.2021.146919 33866172

[B51] Zhang C. Chen X. Wang J. Tan L. (2017). Toxic effects of microplastic on marine microalgae *Skeletonema costatum*: interactions between microplastic and algae. *Environ. Pollut.* 220 1282–1288. 10.1016/j.envpol.2016.11.005 27876228

[B52] Zhang Q. Qu Q. Lu T. Ke M. Zhu Y. Zhang M. (2018). The combined toxicity effect of nanoplastics and glyphosate on *Microcystis aeruginosa* growth. *Environ. Pollut.* 243 1106–1112. 10.1016/j.envpol.2018.09.073 30253301

[B53] Zhang S. Song H. L. Cao X. Li H. Guo J. H. Yang X. L. (2019). Inhibition of methanogens decreased sulfadiazine removal and increased antibiotic resistance gene development in microbial fuel cells. *Bioresour. Technol.* 281 188–194. 10.1016/j.biortech.2019.02.089 30822639

[B54] Zhang Y. Lu J. Wu J. Wang J. Luo Y. (2020). Potential risks of microplastics combined with superbugs: enrichment of antibiotic resistant bacteria on the surface of microplastics in mariculture system. *Ecotoxicol. Environ. Saf.* 187:109852. 10.1016/j.ecoenv.2019.109852 31670243

[B55] Zhao T. Tan L. Huang W. Wang J. (2019). The interactions between micro polyvinyl chloride (mPVC) and marine dinoflagellate *Karenia mikimotoi*: the inhibition of growth, chlorophyll and photosynthetic efficiency. *Environ. Pollut.* 247 883–889. 10.1016/j.envpol.2019.01.114 30731314

[B56] Zheng Y. Li Z. Tao M. Li J. Hu Z. (2017). Effects of selenite on green microalga *Haematococcus pluvialis*: bioaccumulation of selenium and enhancement of astaxanthin production. *Aquat. Toxicol.* 183 21–27. 10.1016/j.aquatox.2016.12.008 27987436

[B57] Zhu Z.-L. Wang S.-C. Zhao F.-F. Wang S.-G. Liu F.-F. Liu G.-Z. (2019). Joint toxicity of microplastics with triclosan to marine microalgae *Skeletonema costatum*. *Environ. Pollut.* 246 509–517. 10.1016/j.envpol.2018.12.044 30583159

