# Supplementary material for: Toxicological Effects of Microplastics and Sulfadiazine on the Microalgae Chlamydomonas reinhardtii
Source: Front Microbiol. 2022 Apr 28;13:865768. doi: 10.3389/fmicb.2022.865768 (PMC9096495; doi:10.3389/fmicb.2022.865768)
Supplement: Supplementary file 1 [file Data_Sheet_1.PDF]

Table S1. Growth inhibition

Intersubjective effect test					
Dependent variable: DATA					
Source	Tape III sum of square	df	Mean square	F	Sig.
Corrected Model	27394.871a	29	944.651	44.067	.000
Intercept	36545.249	1	36545.249	1704.814	.000
SDZ	1899.931	6	316.655	14.772	.000
MP5	10410.192	6	1735.032	80.938	.000
MP1	10240.320	6	1706.720	79.617	.000
SDZ * MP5	964.587	5	192.917	8.999	.000
SDZ * MP1	221.482	5	44.296	2.066	.082
MP5 * MP1	.000	0	.	.	.
a. R Squared = .955 (Adjusted R Squared = .933)					

Post Hoc Tests

		Subset		
SDZ	N	1	2	3
5	9	11.9867		
20	9	15.4222		
10	9	16.7811		
50	9		22.6589	
100	9		24.4244	
200	9		25.5544	
0	36			37.7911
Sig.		.060	.345	1.000

The error term is Mean Square (Error) = 21.437.

a. Uses Harmonic Mean Sample Size = 10.080.

b. The group sizes are unequal. The harmonic mean of the group sizes is used. Type I error levels are not guaranteed.

c. Alpha = 0

S-N-K[a,b,c]

		Subset			
MP5	N	1	2	3	4
10	3	19.8600			
5	3	23.7200	23.7200		
20	3	23.8600	23.8600		
0	54	24.5196	24.5196		
50	21		29.5781		
100	3			38.0667	
200	3				50.0700
Sig.		.486	.284	1.000	1.000

MP1	N	Subset				
		1	2	3	4	5
0	54	20.1457				
5	3		26.8933			
50	21		29.9886			
20	3			38.2033		
10	3			42.4800		
100	3				54.3433	
200	3					69.5133
Sig.		1.000	.346	.194	1.000	1.000

The error term is Mean Square (Error) = 21.437.

a. Uses Harmonic Mean Sample Size = 4.040.

b. The group sizes are unequal. The harmonic mean of the group sizes is used. Type I error levels are not guaranteed.

c. Alpha = 0

The factors of two-way ANOVA for the subsequent analysis were: SDZ (0 = none and 1 = existence); MPs (0 = none, 1 = 1 μm MPs and 2 = 5μm MPs).

Table S2. GSH-Px and SOD activity

Descriptive statistics						
Dependent variable: DATA						
		GSH		SOD		
SDZ	MPS	Mean Difference(I-J)	Std.Error	Mean Difference(I-J)	Std.Error	N
.00	.00	199.8664	7.92018	168.8646	30.87233	6
	1.00	375.4131	212.36219	137.9220	64.72946	6
	2.00	213.1984	34.35104	138.0968	17.93108	6
	Total	262.8259	142.72930	148.2945	42.79286	18
1.00	.00	200.3076	12.04957	203.7842	19.94842	6
	1.00	529.4792	54.71097	456.5810	6.83240	6
	2.00	242.0178	12.24420	507.4044	108.15762	6
	Total	323.9349	153.75641	389.2565	149.12731	18
Total	.00	200.0870	9.72434	186.3244	30.76798	12
	1.00	452.4462	168.32438	297.2515	172.10291	12
	2.00	227.6081	28.82746	322.7506	206.54346	12
	Total	293.3804	149.45849	268.7755	163.16141	36

Intersubjective effect test					
Dependent variable: DATA (GSH-Px)					
Source	Tape III sum of square	df	Mean square	F	Sig.

Corrected model	533680.269[a]	5	106736.054	12.904	.000
Intercept	3098594.186	1	3098594.186	374.612	.000
SDZ	33608.697	1	33608.697	4.063	.053
MPS	459978.889	2	229989.444	27.805	.000
SDZ * MPS	40092.683	2	20046.342	2.424	.106
Error	248144.134	30	8271.471		
Total	3880418.590	36			
Corrected Total	781824.404	35			
a. R Squared = .683（Adjusted R Squared = .630）					

Intersubjective effect test

Dependent variable: DATA (SOD)

Source	Tape III sum of square	df	Mean square	F	Sig.
Corrected model	843721.481[a]	5	168744.296	57.503	.000
Intercept	2600649.599	1	2600649.599	886.222	.000
SDZ	522564.466	1	522564.466	178.074	.000
MPS	126268.494	2	63134.247	21.514	.000
SDZ * MPS	194888.522	2	97444.261	33.206	.000
Error	88036.099	30	2934.537		
Total	3532407.180	36			
Corrected Total	931757.581	35			
a. R Squared = .906（Adjusted R Squared = .890）					

Post Hoc Tests (GSH-Px)

S-N-K[a,b]

MPS	N	Subset	
		1	2
.00	12	200.0870	
2.00	12	227.6081	
1.00	12		452.4462
Sig.		.464	1.000

The Error term is Mean square（Error） = 8271.471

a. Harmonic Mean Difference(I-j) sample size was used = 12.000

b. Alpha = .05

Post Hoc Tests (SOD)

S-N-K[a,b]

MPS	N	Subset	
		1	2
.00	12	186.3244	
1.00	12		297.2515

2.00	12		322.7506
Sig.		1.000	.258
The Error term is Mean square（Error）= 2934.537			
a. Harmonic Mean Difference(I-j) sample size was used = 12.000			
b. Alpha = .05			

Table S3. Chlorophyll

Descriptive statistics

Dependent variable: DATA

		Chl a		Chl b		Chl c		
SDZ	MPS	Mean Difference(I-J)	Std.Error	Mean Difference(I-J)	Std.Error	Mean Difference(I-J)	Std.Error	N
.00	.00	19.3752	1.14472	5.7261	.44095	2.1077	.17239	9
	1.00	15.2606	1.84039	4.5501	.50918	1.6285	.20048	9
	2.00	15.3046	.57739	4.3769	.70938	1.6304	.07413	9
	Total	16.6468	2.32671	4.8843	.81702	1.7889	.27567	27
1.00	.00	16.5577	.84704	4.5716	.22340	1.7737	.09473	9
	1.00	16.0504	1.68177	4.4321	.61847	1.7160	.21222	9
	2.00	16.4146	1.13743	4.2078	.20159	1.8313	.16373	9
	Total	16.3409	1.23954	4.4038	.41094	1.7737	.16483	27
Total	.00	17.9665	1.74803	5.1488	.68397	1.9407	.21850	18
	1.00	15.6555	1.75784	4.4911	.55289	1.6722	.20527	18
	2.00	15.8596	1.04492	4.2923	.51333	1.7308	.16089	18
	Total	16.4938	1.85291	4.6441	.68493	1.7813	.22509	54

Intersubjective effect test

Dependent variable: DATA

Source	Tape III sum of square	df	Mean square	F	Sig.
Chl a					
Corrected model	103.002[a]	5	20.600	12.523	.000
Intercept	14690.535	1	14690.535	8930.104	.000
SDZ	1.263	1	1.263	.768	.385
MPS	58.928	2	29.464	17.910	.000
SDZ * MPS	42.811	2	21.406	13.012	.000
Error	78.963	48	1.645		
Total	14872.499	54			
Corrected Total	181.965	53	a. R Squared = .566（Adjusted R Squared = .521）		
Chl b					

Corrected model	13.424[a]	5	2.685	11.265	.000
Intercept	1164.639	1	1164.639	4886.674	.000
SDZ	3.117	1	3.117	13.079	.001
MPS	7.234	2	3.617	15.177	.000
SDZ * MPS	3.072	2	1.536	6.445	.003
Error	11.440	48	.238		
Total	1189.502	54			
Corrected Total	24.864	53	a. R Squared = .540（Adjusted R Squared = .492）		
Chl c					
Corrected model	1.435[a]	5	.287	11.026	.000
Intercept	171.335	1	171.335	6580.335	.000
SDZ	.003	1	.003	.120	.731
MPS	.717	2	.359	13.776	.000
SDZ * MPS	.715	2	.358	13.730	.000
Error	1.250	48	.026		
Total	174.020	54			
Corrected Total	2.685	53	a. R Squared = .535（Adjusted R Squared = .486）		

Chl a			
S-N-K[a,b]			
MPS	N	Subset	
		1	2
1.00	18	15.6555	
2.00	18	15.8596	
.00	18		17.9665
Sig.		.635	1.000
All groups in the cluster will be displayed Mean Difference(I-J)<br>Based on the measured Mean Difference(I-J)<br>The Error term is Mean square（Error） = 1.645			
a. Harmonic Mean Difference(I-j) sample size was used = 18.000			
b. Alpha = .05			
Chl b			
MPS	N	Subset	
		1	2
2.00	18	4.2923	
1.00	18	4.4911	
.00	18		5.1488
Sig.		.228	1.000
The Error term is Mean square（Error） = .238			

a. Harmonic Mean Difference(I-j) sample size was used = 18.000			
Chl c			
MPS	N	Subset	
		1	2
1.00	18	1.6722	
2.00	18	1.7308	
.00	18		1.9407
Sig.		.281	1.000
The Error term is Mean square（Error）= .026			
a. Harmonic Mean Difference(I-j) sample size was used = 18.000			

Table S4. Photosynthetic efficiency

Descriptive statistics						
Dependent variable: DATA						
		Fv/Fm		YII		
SDZ	MPS	Mean Difference(I-J)	Std.Error	Mean Difference(I-J	Std.Error	N
.00	.00	.6000	.02000	.5333	.01528	3
	1.00	.6000	.01000	.5400	.01000	3
	2.00	.5733	.01155	.5233	.01528	3
	Total	.5911	.01833	.5322	.01394	9
1.00	.00	.6033	.00577	.5367	.00577	3
	1.00	.6200	.01000	.5500	.00000	3
	2.00	.6200	.02000	.5500	.01732	3
	Total	.6144	.01424	.5456	.01130	9
Total	.00	.6017	.01329	.5350	.01049	6
	1.00	.6100	.01414	.5450	.00837	6
	2.00	.5967	.02944	.5367	.02066	6
	Total	.6028	.01994	.5389	.01410	18

Intersubjective effect test					
Dependent variable: DATA					
Source	Tape III sum of square	df	Mean square	F	Sig.
Fv/Fm					
Corrected model	.004[a]	5	.001	4.554	.015
Intercept	6.540	1	6.540	33635.000	.000
SDZ	.002	1	.002	12.600	.004
MPS	.001	2	.000	1.400	.284
SDZ * MPS	.001	2	.001	3.686	.057
Error	.002	12	.000		
Total	6.547	18			
Corrected Total	.007	17			

a. R Squared = .655（Adjusted R Squared = .511）					
YII					
Corrected model	.002[a]	5	.000	2.104	.135
Intercept	5.227	1	5.227	34848.148	.000
SDZ	.001	1	.001	5.333	.040
MPS	.000	2	.000	1.148	.350
SDZ * MPS	.000	2	.000	1.444	.274
Error	.002	12	.000		
Total	5.231	18			
Corrected Total	.003	17			
a. R Squared = .467（Adjusted R Squared = .245）					

Post Hoc Tests

Fv/Fm		
S-N-K[a,b]		
MPS	N	Subset
		1
2.00	6	.5967
.00	6	.6017
1.00	6	.6100
Sig.		.261
All groups in the cluster will be displayed Mean Difference(I-J)<br>Based on the measured Mean Difference(I-J)<br>The Error term is Mean square（Error）= 0		
a. Harmonic Mean Difference(I-j) sample size was used = 6.000		
b. Alpha = .05		
YII		
MPS	N	Subset
		1
.00	6	.5350
2.00	6	.5367
1.00	6	.5450
Sig.		.365
The Error term is Mean square（Error）= 0.000		
a. Harmonic Mean Difference(I-j) sample size was used = 6.000		

Table S5. EPS

Descriptive statistics				
Dependent variable: DATA				
SDZ	MPS	Mean Difference(I-J)	Std.Error	N
.00	.00	27.8267	9.78952	6
	1.00	45.5600	14.54978	6

	2.00	47.0267	8.60295	6
	Total	40.1378	13.88658	18
1.00	.00	28.5600	8.03194	6
	1.00	52.2267	18.79135	6
	2.00	83.4267	21.74826	6
	Total	54.7378	28.22272	18
Total	.00	28.1933	8.54585	12
	1.00	48.8933	16.39675	12
	2.00	65.2267	24.69793	12
	Total	47.4378	23.13781	36

Intersubjective effect test

Dependent variable: DATA

Source	Tape III sum of square	df	Mean square	F	Sig.
Corrected model	12376.769[a]	5	2475.354	11.675	.000
Intercept	81012.339	1	81012.339	382.087	.000
SDZ	1918.440	1	1918.440	9.048	.005
MPS	8266.942	2	4133.471	19.495	.000
SDZ * MPS	2191.387	2	1095.693	5.168	.012
Error	6360.773	30	212.026		
Total	99749.882	36			
Corrected Total	18737.542	35			

a. R Squared = .661（Adjusted R Squared = .604）

Post Hoc Tests

S-N-K[a,b]

MPS	N	Subset		
		1	2	3
.00	12	28.1933		
1.00	12		48.8933	
2.00	12			65.2267
Sig.		1.000	1.000	1.000

All groups in the cluster will be displayed Mean Difference(I-J)
Based on the measured Mean Difference(I-J)
The Error term is Mean square（Error）= 212.026

a. Harmonic Mean Difference(I-j) sample size was used = 12.000

b. Alpha = .05
